# Applying Contemporary Immunology to Elucidate Heterologous Effects of Infant Vaccines and to Better Inform Maternal–Infant Immunization Practices

**DOI:** 10.3389/fimmu.2015.00064

**Published:** 2015-02-16

**Authors:** Christopher B. Wilson

**Affiliations:** ^1^Discovery & Translational Sciences, Global Health Program, Bill & Melinda Gates Foundation, Seattle, WA, USA

**Keywords:** infant immunity, trained immunity, heterologous immunity, maternal immunization

“Without knowledge action is useless, and action without knowledge is futile”

Abu Bakr, 547 CE

This series of publications “*Understanding the Ontogeny of the Immune System to Promote Immune-Mediated Health for Life*” in Frontiers in Immunology provides a timely summary of the many recent advances in knowledge regarding the immune response of the mother, fetus, newborn, and young infant. This basic information provides the foundation from which new approaches to foster a smooth transition from the relatively insular and protected *in utero* environment – where bidirectional tolerance between mother and fetus is the over-arching goal – to the postnatal environment – where tolerance to self and the acquired microbiota must be balanced against the threat of pathogen invasion. But to truly address the theme inherent in the title of the Immunotherapies and Vaccines section of Frontiers in which these publications appear, knowledge is only the beginning. As Abu Bakr implied, the far greater (and more important) challenge is for immunologists to bridge between the separate siloes in which they and vaccinologists traditionally operate, then together to work effectively with microbiologists, systems, computational and structural biologists, bioengineers, and formulation chemists to translate emerging biological knowledge into new products and solutions to further reduce the global burden of infectious diseases.

Although there has been a gratifying 49% decrease since 1990 in global mortality under the age of 5 years to a rate of ~6.3 million/year in 2013, the rate remains too high; moreover, the rate of decline in neonatal mortality has been less (40%), with the result that 45% of all under five mortality now occurs in the first month of life (http://data.unicef.org/child-mortality/). Collectively, preterm birth and infection are believed to account for approximately one-third and 20% of these neonatal deaths, respectively, with a fraction of preterm birth itself related to infection. These numbers indicate the remaining unmet need to more effectively and efficiently apply existing vaccines and to develop new vaccines to further reduce the burden of infectious diseases in early life.

Active immunization of infants and young children – along with improvements in hygiene and nutrition – appears to have been an important factor in this reduction in under-five mortality. And while extending these benefits of vaccines more broadly throughout the world and developing new vaccines against major pathogens for which effective vaccines are not yet available are clear priorities going forward, this Opinion will focus on knowledge gaps in two areas, which if addressed could inform approaches by which to further reduce under-five mortality through immunization. The first need is to better define the heterologous effects of vaccines and vaccine schedules in humans and thereby to provide an evidence-base from which vaccine regimens might be further optimized to protect against target pathogens, while promoting potential for heterologous benefits where possible. The other gap lies in the knowledge needed to design a harmonized program of maternal and infant immunization to protect newborns and young infants from diseases in addition to tetanus, such that protective antibodies acquired by the infant through immunization of the mother do not unduly impede the responses of the infant to those vaccines.

Beginning with the pioneering studies by Mackeness in the 1960s, an extensive body of research from mouse and other model systems has shown that immunization with BCG, infection with heterologous pathogens, and exposure to microbial products can “non-specifically” increase, and in other contexts decrease, host resistance to other infectious agents, cancer, and autoimmunity (1–3). Similarly, the impact of antibodies acquired from the mother on postnatal immunization of their infants has been evaluated in model systems, as reviewed in this series by Niewisk (4). This information provides supporting biological rationale and illustrates principles that are likely relevant to humans. Herein, the focus is on human evidence and evidence gaps, which if addressed would illuminate the degree to which those principles apply to human infants and inform ways be which they might be translated into appropriate action.

## Heterologous Effects of Vaccines

The immune system is designed to continuously monitor environmental context. On association with the microbial world during and after parturition, both the innate and adaptive immune systems must rapidly learn from and establish beneficial equipoise with their new microbe-rich world. Whereas, it was previously thought that learning took place only within the adaptive (antigen-specific) immune system, early clues, and a wealth of more recent data indicate that the innate immune response can also be trained to respond differently on subsequent encounters, though likely without the extended durability of immune memory characteristic of long-lived, adaptive immune responses. As noted by MacGillivray and Kollmann (5) and described in additional detail in other publications (6–8), such “trained” immunity and its potential impact on health outcomes appear to have been hiding in plain sight for some time in the form of non-specific or heterologous effects of microbial exposure or immunization on near-term risk of death – apparently death caused by infections other than those in the “training” microbes or vaccines.

An increasing body of ecological evidence supports the notion that live vaccines, most notably BCG and measles vaccines, reduce risk of near-term death, whereas no such benefit and potentially increased risk have been ascribed to inactivated vaccines, such as DTP (6). Consistent with the less durable nature of “trained” innate immunity, extant evidence suggests that the most recently received vaccine is deterministic – if a live vaccine, risk is decreased, if inactivated, risk is increased in the following months. In addition to this ecological record, a composite analysis of two randomized controlled trials of low birth weight infants in Guinea-Bissau in which BCG was given at birth or delayed until a later age revealed that birth administration was associated with a 58, 48, and 21% reduction in infant mortality, respectively, in the first days, month, and year of life, apparently related to reduced rates of sepsis and respiratory illness (6). MacGillivray and Kollman (5) also refer to a report from Australia indicating that BCG given at birth may enhance the immune response to routine immunizations during the first 6 months of life. However, only the quantity of antibody to two pneumococcal serotype was significantly greater in those given BCG at birth. And there was an inconsistency in this study compared to a previous one from the Gambia, in that the antibody response to the birth dose of hepatitis B vaccine was lower in those given concurrent BCG in Australia and higher in the Gambia (9). That said, there is considerable biological plausibility that immunization, like other environmental exposures, can have heterologous effects on the immune response in ways that affect clinical outcomes. For example, it was recently reported that priming through TLR5 by flagellin on gut commensal bacteria is an important factor in the ability of mice to generate robust immune responses to a parenterally administered viral (influenza) vaccine (10).

Biological plausibility and the body of largely ecological evidence notwithstanding, more rigorous controlled human trials and contemporary analytical approaches to probe the underlying biological mechanisms for the benefits, risks, or lack of heterologous effects of vaccines are needed. The most striking apparent heterologous benefit of BCG and measles vaccination is reduction in mortality, yet for the most part, the causes of that mortality are wholly or at best partly cryptic. New approaches to employ minimally invasive autopsies in which small samples are fully analyzed to detect known pathogens, unknown, and unculturable potential pathogens or pathobionts and in parallel to assess the human immune response in molecular detail and at single cell resolution may illuminate causes and inform more appropriately targeted next steps. The import of such studies goes far beyond academic interest, because such information is needed to provide a mechanism-based rationale for the clinical evaluation and optimization of vaccine administration schedules.

## Toward a Harmonized Maternal and Infant Immunization Program

While heterologous immunity from a birth dose of BCG may provide a broad measure of protection for the first weeks to months of life, a complementary and more targeted approach is immunization of the pregnant woman to induce antibodies and thereby passively immunize her as yet unborn infant, as highlighted in the review by Niewisk (4). This approach has only been practiced widely for prevention of neonatal tetanus. But more recently, administration of combination of tetanus, diphtheria, and acellular pertussis vaccine boosters has led to the reduction of infant deaths due to pertussis in the United Kingdom [reviewed in Ref. (11)]. This approach has been implemented in other developed countries but not yet in resource-limited settings due in part to the higher cost and less durable immunity provided by acellular compared to whole cell pertussis vaccines. The observation that pregnant women were a highly vulnerable population during the recent H1N1 influenza pandemic, prompted broader implementation of maternal influenza vaccination. The value of this approach to protect both the mother and her newborn infant was demonstrated recently in South Africa – maternal immunization resulted in efficient transplacental transfer of influenza antibodies and reduced the rate of confirmed influenza illness in the infant by approximately half; this reduction appeared to reflect both direct protection of infants by the passively derived antibody and reduced risk for infection of the mother and subsequent transmission to her infant (12).

These successes and the absence of major vaccine-attributable adverse events have created momentum to more broadly implement maternal immunization to protect infants in the first 3–4 months of life before immunization of the infant would provide protection. Vaccines for respiratory syncytial virus (RSV), cytomegalovirus (CMV), and the group B streptococcus (GBS), which are all under active development, would when available logically be included in maternal immunization programs. In the case of CMV and GBS, where death and disability are largely restricted to infection acquired *in utero* or the first 3 months of life, respectively, maternal immunization without infant immunization is all that would be required.

There is a potential caveat regarding the benefits of maternal immunization – inhibition by passively acquired maternal antibodies of the infant’s immune response to active immunization with homologous vaccines [reviewed in Ref. (4, 13)]. This inhibition has been clearly demonstrated in the context of measles immunization – maternally derived antibody appears to impede the infant’s antibody but not T cell responses. Despite the inhibition of antibody production, B cell priming appears to occur in the infant, which allows high-affinity, protective anamnestic antibody responses to booster measles immunization given at a later age (13), even though the final antibody concentrations may be somewhat lower (14).

What is the mechanism for the inhibitory effect of maternal antibody? Niewisk (4) makes a strong case that the dominant mechanism is inhibitory signaling through FcγRIIb. This conclusion is consistent with the retention of T cell responses and a wealth of experimental evidence on Fcγ receptor and B cell biology (15). Because FcγRIIb-mediated inhibition of B cell responses is a general mechanism, which in the absence of passive antibody serves both as a rheostat to prevent excessive antibody production and to foster affinity maturation (15), interference by passively acquired antibodies should be a universal phenomenon for all vaccines. Evidence cited by Niewisk (4) suggests that this is true though the degree and consistency of inhibition observed in humans varies between vaccines and studies (11, 13). This lack of consistency and the paucity of direct mechanistic evidence in humans impede implementation of a rational, harmonized, passive maternal, and active infant immunization program.

Until recently, the technologies and cross-disciplinary scientific cooperation needed to provide such evidence were lacking. However, technology is now in hand to analyze the human antibody, B and plasma cells and associated T helper cell repertoires, and responses on small volumes of blood and thereby to trace over time the evolutionary pathways of human infant immune responses to vaccines at single cell, molecular, and structural levels. Such studies have illuminated the pathways by which broadly neutralizing antibodies are induced in HIV-infected individuals (16, 17) and led to the identification of antibodies that can neutralize both RSV and the related human metapneumovirus (18). Elucidation of the structure of the RSV F protein in complex with these and other neutralizing monoclonal antibodies has led to the structure based-design of recombinant F protein vaccines stabilized or scaffolded in a pre-fusion confirmation, which induce protective antibodies in non-human primates (19, 20). Such information could be used to design different RSV vaccines for maternal immunization and infant immunization (e.g., one in a pre-fusion and another in a post-fusion confirmation), such that vaccine-induced maternally derived antibodies would not bind to and inhibit the antibody response to the infant vaccine. Alternatively, advances in monoclonal antibody isolation, in engineering of antibodies to enhance duration and potency, and in manufacturing technologies to reduce production costs might together be harnessed to produce monoclonal antibodies by which to passively protect newborn infants [e.g., for RSV or RSV and metapneumovirus (18)], and thereby eliminate the need to immunize the mother. In either scenario, the later need to actively immunize the infant and potential inhibition by passively derived antibodies must be addressed (unless the duration of passive protection is sufficient to encompass the period of risk, as for GBS). In addition to the possible engineering of infant immunogens that are not engaged by passively derived antibodies as described above for RSV, vaccine antigen content and formulation may be optimized through the use of adjuvants that more effectively yet safely promote antibody responses in young infants, as was shown for the MF59-adjuvanted H1N1 vaccine (21), which may allow inhibitory effects of maternal antibodies to be overcome (4); this optimization may be usefully informed by a more comprehensive knowledge of the infant’s age-dependent innate immune responses (5, 22). Such approaches, and the translational and clinical research programs needed to evaluate them, could form the basis for a more evidence-based approach to vaccine design, formulation, and immunization schedules for mother and infant.

## Conflict of Interest Statement

The author declares that the research was conducted in the absence of any commercial or financial relationships that could be construed as a potential conflict of interest.
